# Aspiration Pneumonia and Difficult Removal of an Exfoliated Primary Tooth—A Case Report

**DOI:** 10.1002/ccr3.72300

**Published:** 2026-03-17

**Authors:** Sarah Winterland, Dennis Nordhoff, Thomas Boesing, Eckard Hamelmann, Stephan Winterland

**Affiliations:** ^1^ Department of Paediatrics Bielefeld University, University Hospital OWL, Children's Center Bethel Bielefeld Germany; ^2^ Department of Neonatology and Pediatric Intensive Care Medicine Bielefeld University, University Hospital OWL, Children's Center Bethel Bielefeld Germany

**Keywords:** accidental, aspiration pneumonia, bronchoscopy, exfoliated primary tooth

## Abstract

The aspiration of exfoliated primary teeth is extremely rare yet presents a high risk for aspiration pneumonia. This is the first reported case in a child with no underlying conditions. Expertise in the removal of foreign bodies is vital for an optimal treatment outcome and should be performed at specialist centers.

## Graphical abstract

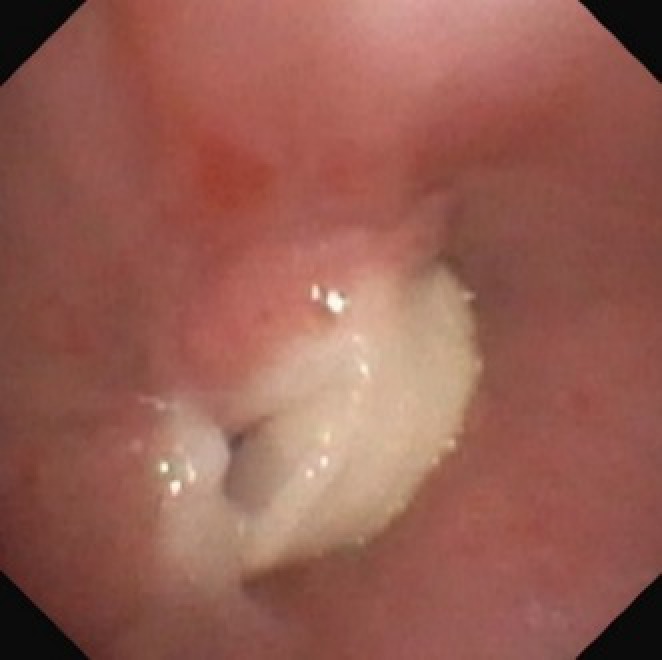

**Abbreviations**
CXR: chest x‐ray
FB: Foreign body
FBA: Foreign body aspiration

## 1 Introduction/Background

Foreign body aspiration (FBA) is a common cause of morbidity and mortality in children [1, 2]. According to Salih et al., 93.8% of cases are below 16 years of age, and 77.8% are below 3 years of age [2]. Boys are more often affected than girls [3]. Aspirated foreign bodies are classified as food or nonfood substances [1, 2, 4] and show geographical and global variation [2]. A Belgian study showed that 91% of aspirated substances in children were organic in nature and more than half were peanuts [5]. Organic substances show a higher rate of complication due to their ability to absorb moisture, possibly resulting in postobstructive pneumonia [1]. Another reason for higher rates of complication in this group is the fact that organic substances may elicit a strong inflammatory response within the bronchial tissue, impeding their removal [1, 6].

The aspiration of teeth is rare. Thus, in only 0.4% of foreign body aspirations a tooth was the aspirated substance [7]. Dental aspiration often occurs after maxillofacial trauma or oral surgery [7]. Other cases are found in patients with neurological handicaps [7]. The aspiration of exfoliated primary teeth, however, is to be considered extremely rare as to our knowledge only 4 cases have been reported thus far [8, 9, 10, 11]. The case presented here is to our best knowledge the first where no underlying conditions were to be noted.

## 2 Case History

We present the case of a 7‐year‐old German female. The patient has no preexisting conditions or allergies. She receives no long‐term medication and is fully vaccinated.

The patient was admitted to our emergency room late in the evening. According to the patient and her parents, she had accidentally swallowed one of her milk teeth 3 days prior to admission. After the incident, the patient immediately began coughing, which suspended spontaneously after a short while. Eight hours later, the patient developed a fever and over the course of the next 3 days, a cough and dyspnoea. Food and fluid intake were also affected and reduced. The combination of these symptoms led to the admission to our emergency department.

On admission, the patient's weight was 26 kg. She had a fever of 39.1°C and her oxygen saturation was 96%. The physical examination revealed a slightly reduced general condition with no cognitive impairment. The tonsils were slightly enlarged and a white coating of the tongue could be observed. The auscultation of the lungs showed distinctly reduced respiratory sounds over the right lung and subtly reduced respiratory sounds over the basal part of the left lung. No rhonchus or wheezing could be heard. The remaining physical examination showed no reportable abnormalities. Notably, no signs of sepsis were present.

## 3 Methods (Differential Diagnosis, Investigations and Treatment)

Because of the patient's personal history and her physical examination, aspiration pneumonia with a possible pleural effusion was the most likely diagnosis.

Blood works including a blood gas analysis and blood culture were performed on admission, showing leucocytosis (14.7/nL) and greatly elevated CRP values (282 mg/L) as well as a slightly reduced hemoglobin level (11.2/nL). The blood gas analysis revealed a slight respiratory alkalosis. The remaining blood tests revealed no abnormalities.

Continuous monitoring via ECG and oxygen saturation was established. Intravenous fluids were administered at initially 1950 mL/kg/day and antibiotic therapy with ampicillin‐sulbactam (16.2 mg/kg ampicillin content, 3 times a day) was begun—the calculated standard of care at our facility in conjunction with national treatment guidelines for aspiration pneumonia. The patient received analgesics on demand (ibuprofen and paracetamol) and supportive breathing therapy.

**FIGURE 1:**
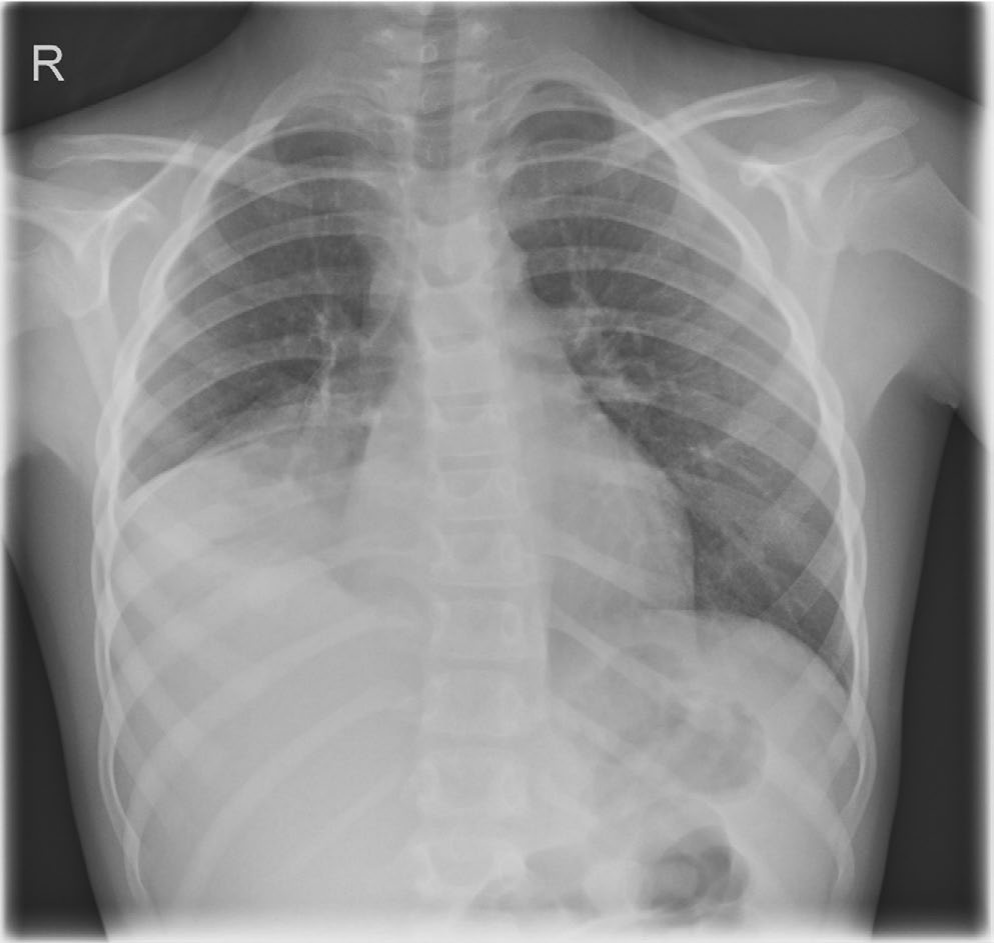
Chest X‐Ray on Admission (day 3 after aspiration event).

Directly after admission a chest X‐Ray was performed, showing consolidation in the right lower lobes as part of a suspected superimposed image of infiltrates and atelectasis (see Figure 1) confirming the initial clinical diagnosis of (aspiration) pneumonia. A small pleural effusion on the right side could not be ruled out in this manner but was later disproved via ultrasound.

**FIGURE 2:**
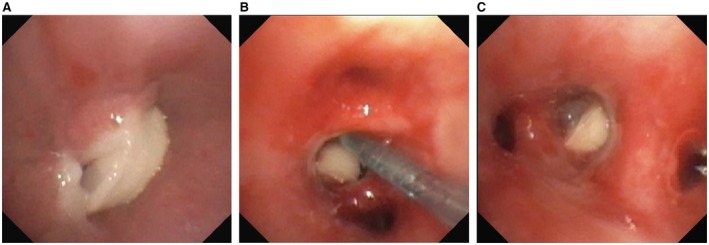
(A–C) Endoscopic images of the foreign object prior to removal (day 4 after aspiration event).

The next morning, removal of the suspected aspirated milk tooth was attempted by bronchoscopy. Initially, a flexible bronchoscope (Olympus BF‐P190, 4.2 mm outer diameter; Olympus Europa SE & Co. KG, Hamburg) was used to get a first overview under propofol sedation and spontaneous ventilation with continuous vital parameter monitoring (HR, SpO2, BP, etCO2, BR). No abnormalities could be identified in the oropharynx up to the epiglottis. At the entrance to the trachea, a lot of white, viscous mucus could be seen. In the outflow of the right intermediate bronchus, what appeared to be a white foreign object could be identified. However, removal using the flexible technique proved impossible as the forceps were only able to grasp mucus. Therefore, our approach changed. A muscle relaxant (rocuronium in fractional amounts, total dose 2 mg/kg) was administered additionally, the patient was intubated (5.0 blockable tubus, Portex; ICU medical Germany GmbH, Lüdenscheid), and the use of a rigid bronchoscope (Karl Storz bronchoscope tube according to DOESEL‐HULZY, 4 mm outer diameter; Karl Storz SE & Co. KG, Tuttlingen) simultaneously to a smaller flexible instrument (Olympus BF‐XP190, 3.1 mm outer diameter; Olympus Europa SE & Co. KG, Hamburg) was implemented. Using this combination, the before‐suspected foreign object was identified as an accumulation of highly viscous mucus, which was successfully suctioned out and sent to the lab for further microbiological analysis. After having removed this obstacle, the real foreign object could be identified in the lateral basal segment of the right lung (segment 9) (see Figure 2A–C).

Due to the fact that the foreign object had been aspirated this deep into the bronchial system, the optical pliers could not reach it. Several attempts with longer flexible tongs were unsuccessful as well, as the foreign object was blocked into position causing the tongs to slip off at each try. Further different removal methods were unsuccessful: positioning a Fogathy catheter behind the foreign object, using a barrel bag or using longer optical tongs. Success was at last achieved by using long tongs and very carefully positioning them parallel to the minuscule opening slits remaining on each side of the foreign object after having sucked off all visible mucus. Due to the difficult removal, small bleedings occurred that were stopped by local application of tranexamic acid and adrenalin. A therapeutic bronchial lavage was performed and 2 lavage specimens were sent for cultural testing (culture‐bacterial method: cultivation and propagation on solid (agar) culture medium; qualitative determination). On careful inspection, only a minor traumatisation of the bronchial mucosa was notable after removal of the foreign body. In the final assessment, no anatomical abnormalities were found in the entire bronchial tree. Hence, neither fluoroscopy nor any other additional imaging was used during the whole procedure. The procedure took about 2 h.

Afterwards the patient remained intubated in order to open up the atelectasis. On the next day (2 days after admission) the patient was successfully extubated and subsequently received supportive ventilation via high flow nasal cannula for one additional day. Hereafter the patient continued to breathe spontaneously and without help. The already initiated antibiotic therapy was continued until discharge. No change in antibiotic regime was necessary as the lavage samples taken during bronchoscopy showed growth of haemophilus influenza sensitive to ampicillin‐sulbactam as well as amoxicillin and clavulanic acid with negative culture results being available within 2 days after bronchoscopy.

After removal of the tooth the initial X‐Ray was reevaluated. With the knowledge of the prior events a clouding with a diameter of about 5 mm was identified at the height of the right seventh rib, believed to be the projection of the aspirated tooth.

**FIGURE 3:**
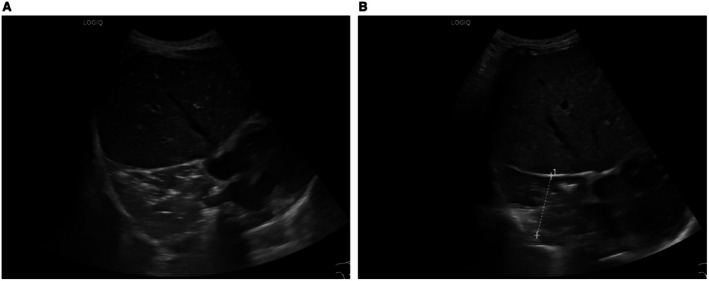
(A) Pleural ultrasound showing atelectasis 1 day post removal (day 5 after aspiration event). (B) Pleural ultrasound showing atelectasis 3 days post removal (day 7 after aspiration event).

A chest X‐Ray was not performed after the tooth was removed; instead, to reevaluate the extent of the known atelectasis and of our therapeutic success, two ultrasounds of the pleura were performed (see Figure 3A,B). In the second ultrasound 3 days after the tooth's removal, the atelectasis had shrunken significantly but had not yet disappeared completely.

The patient's blood work improved quickly, with leucocytes in normal range 2 days after removal and CRP levels dropping while still being elevated (132 mg/L). The result of the blood culture was also negative.

Consistent with these improvements, the patient's general condition also ameliorated and her temperature returned to normothermia 3 days after bronchoscopy.

The patient was discharged in a significantly improved general condition 3 days after the successful removal of the tooth. No prior transfer to a peripheral ward took place. The patient was instructed to continue her antibiotic therapy with amoxicillin and clavulanic acid. (29.8 mg/kg, amoxicillin content, 3 times a day) orally for 4 days. In addition to that, a follow‐up X‐Ray was advised 2 weeks after discharge.

### 3.1 Case Timeline

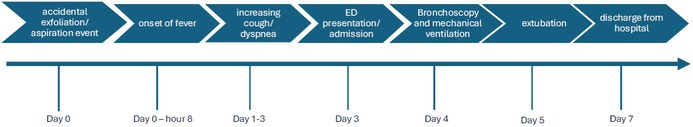

## 4 Conclusions and Results (Outcome and Follow‐Up)

Even though a follow‐up X‐Ray was advised at discharge, the patient did not return to our hospital. Therefore we can unfortunately not report on any of the expected general imaging improvements. During the drafting of this case report the family was contacted twice via telephone to gain consent for publishing 3 and, again, 11 months after the aspiration event. During these phone calls it was revealed that the patient had been in very good general condition since the discharge. Notably, no symptoms of pneumonia (fever, dyspnoea, and cough) or signs of long‐term consequences (wheezing, recurrent lower airway infections) were to be reported. There were no further incidents of witnessed or suspected aspiration. The patient's parents stated that the good general condition was the reason no follow‐up X‐ray was performed at our facility and no medical consultation with a local primary care physician was sought.

## 5 Discussion

Aspiration of exfoliated primary teeth is very rare and has thus far only been described four times [8, 9, 10, 11]. Yet in all the herebefore mentioned cases the patient had some kind of preexisting condition, making this the first reported case of an accidental aspiration of an exfoliated primary tooth in a patient with no preexisting diseases [8, 9, 10, 11]. The hereby described patient did not fall under a high‐risk category concerning age, sex or comorbidities, and admission to our emergency department took place with a significant time gap of 3 days after the incident. Therefore without a careful and complete anamnesis the aspiration could have been missed and treatment could therefore have been delayed. It is also notable that patients (and caregivers) often do not recall aspiration events, impeding an accurate anamnesis [1]. Therefore, it is pivotal in our opinion not to rule out a foreign body aspiration due to the lack of a conclusive history or a patient not belonging to a typical risk category.

Passàli et al. state that “[c]linical signs of FB inhalation have low positive predictive values” and that therefore every tool available should be used to confirm or rule out the diagnosis [3]. Thus, fewer than 40% of patients show the classical symptoms of FB inhalation [12]. For patients with any unambiguous history or in an unstable condition (rigid), bronchoscopy is the primary key pillar in diagnostics as well as therapy [1]. In all other cases, other diagnostical tools should be considered; however, in case of any doubt, bronchoscopy should always be performed to rule out FBA, as this is highlighted in particular in the national German guideline for endoscopy of the airway in childhood [13]. For example a chest X‐Ray is easily obtained yet may or may not be useful as not all FB can be identified here [1, 3] and a normal chest X‐Ray is found in up to 20% of cases of FBA [14]. Most organic substances are to be considered radiolucent [1]. Despite this, teeth can be directly identified in X‐Ray pictures, as seen for example in the case report published by Amini and Boyton [8], yet in our case, due to the location of the FB and to other abnormalities (atelectasis and mucus plugging) in the X‐Ray, it was missed on first examination as these pathologies can mimic consolidations of teeth. Therefore, in conjunction with the German national guideline on “Interdisciplinary care for children after foreign body aspiration or foreign body ingestion”, bronchoscopy was the consistent next step in diagnosis and therapy.

Treatment of FB aspiration in children for decades consisted mainly of rigid bronchoscopy [12]. In recent years flexible bronchoscopy more frequently becomes the first‐line tool in these cases as improvements in the area of anesthesia enabled the use of larger flexible bronchoscopes and thus the use of multiple forceps [15]. Advantages of this change in regime are the better visualization of FBs and the possibility to better remove FBs beyond the main bronchi [15]. In addition to that, small fragments of FBs can be missed during rigid bronchoscopy [15]. In the case presented here, we chose to initially perform flexible bronchoscopy; yet, when unsuccessful in the removal of the tooth, we changed to rigid bronchoscopy. This demonstrates that while it is important to be aware of current treatment regimens and guidelines, treatment of FB inhalations always needs to be considered on a case‐to‐case basis. In our opinion, an extraction plan based on medical imaging should be made before performing bronchoscopy, if possible. In our case, this was unfortunately unfeasible as the tooth in the X‐Ray was only identified after the extraction. Additionally, changes in approach should be applied where necessary, and all available tools should be considered during extraction. The case here described shows that extraction of foreign bodies from the airways can be highly demanding and requires a high level of expertise and improvisation.

In all other reported cases as well as the one presented here, the patients developed an infection as a complication of the aspiration [8, 9, 10, 11]. In our case as well as in three others, aspiration pneumonia was observed [9, 10, 11]. In the remaining case, the patient developed postobstructive purulent bronchitis [8]. It is to be said that in none of the cases mentioned above was medical help sought for directly after the incident. If removal of the tooth had happened at an earlier time, these complications could have most likely been prevented. Yet it is also of note that symptoms of pneumonia, in our case revealed by the onset of fever, were present fairly quickly after the initial incident, with an interval of only 8 h.

As stated our follow‐up is limited due to the fact that the family did not seek out the recommended follow‐up CXR. Therefore we cannot entirely rule out any lasting consequences.

We conclude that, even though aspiration of exfoliated primary teeth is extremely rare, it is of utmost importance to accurately diagnose and swiftly remove the aspirated tooth; as otherwise complications quite likely will occur:

- Consider the differential diagnosis of foreign body aspiration in patients with indicative clinical findings when dentition change occurs.
- Management of aspirated teeth should lay in experienced hands in centers fully equipped for pediatric bronchoscopy.
- Combined flexible and rigid bronchoscopy can be necessary for deep or difficult foreign bodies.

## Author Contributions

**Sarah Winterland:** conceptualization, investigation, methodology, writing – original draft, writing – review and editing. **Dennis Nordhoff:** investigation, writing – review and editing. **Thomas Boesing:** investigation, writing – review and editing. **Eckard Hamelmann:** writing – review and editing. **Stephan Winterland:** conceptualization, investigation, supervision, writing – review and editing.

## Funding

We acknowledge support for the publication costs by the Open Access Publication Fund of Bielefeld University and the Deutsche Forschungsgemeinschaft (DFG).

## Ethics Statement

The authors have nothing to report.

## Consent

Written informed consent was obtained from the patient and her guardians for publication of this case report and any accompanying images. A copy of the written consent is available for review by the Editor‐in‐Chief of this journal.

## Conflicts of Interest

The authors declare no conflicts of interest.

## Data Availability

Data sharing not applicable to this article as no datasets were generated or analyzed during the current study.

## References

[1] D. Bajaj , A. Sachdeva , and D. Deepak , “Foreign Body Aspiration,” Journal of Thoracic Disease 13, no. 8 (2021): 5159–5175, 10.21037/jtd.2020.03.94. 34527356 PMC8411180

[2] A. M. Salih , M. Alfaki , and D. M. Alam‐Elhuda , “Airway Foreign Bodies: A Critical Review for a Common Pediatric Emergency,” World Journal of Emergency Medicine 7, no. 1 (2016): 5–12, 10.5847/wjem.j.1920-8642.2016.01.001. 27006731 PMC4786499

[3] D. Passàli , M. Lauriello , L. Bellussi , G. C. Passali , F. M. Passali , and D. Gregori , “Foreign Body Inhalation in Children: An Update,” Acta Otorhinolaryngologica Italica: Organo Ufficiale Della Societa Italiana di Otorinolaringologia e Chirurgia Cervico‐Facciale 30, no. 1 (2010): 27–32. 20559470 PMC2881610

[4] Centers for Disease Control and Prevention (CDC) , “Nonfatal Choking‐Related Episodes Among Children—United States, 2001,” MMWR. Morbidity and Mortality Weekly Report 51, no. 42 (2002): 945–948. 12437033

[5] F. Baharloo , F. Veyckemans , C. Francis , M. P. Biettlot , and D. O. Rodenstein , “Tracheobronchial Foreign Bodies: Presentation and Management in Children and Adults,” Chest 115, no. 5 (1999): 1357–1362, 10.1378/chest.115.5.1357. 10334153

[6] I. S. Sehgal , S. Dhooria , B. Ram , et al., “Foreign Body Inhalation in the Adult Population: Experience of 25,998 Bronchoscopies and Systematic Review of the Literature,” Respiratory Care 60, no. 10 (2015): 1438–1448, 10.4187/respcare.03976. 25969517

[7] Z. Xu , L. Wu , and Z. Chen , “Dental Aspiration in a Pediatric Patient: A Case Report,” Journal of International Medical Research 51, no. 12 (2023): e3000605231215220, 10.1177/03000605231215220. PMC10693794 38041829

[8] H. Amini and J. R. Boynton , “Aspiration of an Exfoliated Primary Molar in a Child With Cerebral Palsy: A Case Report,” Special Care in Dentistry: Official Publication of the American Association of Hospital Dentists, the Academy of Dentistry for the Handicapped, and the American Society for Geriatric Dentistry 42, no. 4 (2022): 416–420, 10.1111/scd.12686. 34874069

[9] M. Park , K.‐E. Lee , and J.‐H. Lee , “Pneumonia due to Tooth‐Like Foreign Body Aspiration in a Child With Seizure Disorder,” Journal of Korea Assosiation for Disability and Oral Health 14, no. 1 (2018): 26–30, 10.12655/KADH.2018.14.1.26.

[10] R. Steelman , E. Millman , M. Steiner , and R. Gustafson , “Aspiration of a Primary Tooth in a Patient With a Tracheostomy,” Special Care in Dentistry: Official Publication of the American Association of Hospital Dentists, the Academy of Dentistry for the Handicapped, and the American Society for Geriatric Dentistry 17, no. 3 (1997): 97–99, 10.1111/j.1754-4505.1997.tb00876.x. 9582711

[11] H. Suzuki , T. Hiraoka , M. Mizumoto , and Y. Kondo , “Pediatric Case of Exfoliated Primary Tooth Aspiration,” Pediatrics International: Official Journal of the Japan Pediatric Society 64, no. 1 (2022): e15261, 10.1111/ped.15261. 35938601

[12] N. Saki , S. Nikakhlagh , F. Rahim , and H. Abshirini , “Foreign Body Aspirations in Infancy: A 20‐Year Experience,” International Journal of Medical Sciences 6, no. 6 (2009): 322–328, 10.7150/ijms.6.322. 19851473 PMC2764343

[13] Gesellschaft für Pädiatrische Pneumologie, Gesellschaft für Neonatologie und Pädiatrische Intensivmedizin, Deutsche Gesellschaft für Kinder‐ und Jugendmedizin, Österreichische Gesellschaft für Pneumologie, Arbeitsgemeinschaft Pädiatrische HNO‐Heilkunde der Deutschen Gesellschaft für Hals‐Nasen_Ohren‐Heilkunde, Kopf‐Hals‐Chirurgie, Deutsche Gesellschaft für Anästhesiologie und Intensivmedizin mit dem Wissenschaftlichen Arbeitskreis Kinderanästhesie and D. Schramm , “Atemwegsendoskopie im Kindesalter: AWMF‐Register Nr.026/025” (2020), https://www.paediatrische‐pneumologie.eu/.

[14] M. B. Ramos , M. Botana‐Rial , E. García‐Fontán , A. Fernández‐Villar , and M. G. Torreira , “Update in the Extraction of Airway Foreign Bodies in Adults,” Journal of Thoracic Disease 8, no. 11 (2016): 3452–3456, 10.21037/jtd.2016.11.32. 28066626 PMC5179474

[15] S. Louhaichi , N. Boubaker , B. Hamdi , et al., “Removal of Airway Foreign Body Using Flexible Bronchoscopy in Children,” Archives de Pediatrie: Organe Officiel de la Societe Francaise de Pediatrie 31, no. 4 (2024): 264–269, 10.1016/j.arcped.2024.01.008. 38637247

